# Study of the Antagonism of Biocontrol Strains Against the Blue-Stain Fungus of Rubberwood

**DOI:** 10.3390/jof11010055

**Published:** 2025-01-12

**Authors:** Xiaolong Wu, Susu Yang, Jun Li, Jian Qiu, Lei Qin

**Affiliations:** 1 Yunnan Provincial Key Laboratory of Wood Adhesives and Glued Products, Southwest Forestry University, Kunming 650224, China; wxl2055755657@126.com (X.W.); 18864260830@163.com (S.Y.); 13116271761@163.com (J.L.); 18987137382@163.com (J.Q.); 2 College of Materials and Chemical Engineering, Southwest Forestry University, Kunming 650224, China

**Keywords:** rubberwood, blue-stain fungus, endophytic fungi, biological control, antagonism

## Abstract

Wood blue staining is one of the most common wood defects, which commonly occurs in rubberwood and Masson pine. It not only affects the appearance of the wood, but also its properties. In this study, rubberwood from Xishuangbanna was examined. During the process, blue-stain fungi and endophytic fungi were isolated and identified. The antagonism of biocontrol strains against blue-stain fungi was studied, and their inhibitory effects were evaluated by inoculating the biocontrol strains on non-blue-stained rubberwood. The morphology and distribution of the strains on the wood were observed using polarized light and fluorescence microscopy, infrared spectroscopy, scanning electron microscopy (SEM–EDS), and X-ray diffraction (XRD). The effects of biocontrol strains on the blue stain of the wood were then evaluated. The results showed that at room temperature, the fungus causing the blue stain in rubberwood was identified as *Lasiodiplodia theobromae*, and the biocontrol strains of endophytic fungi isolated were *Trichoderma koningii* and *Trichoderma reesei*. It was found that *T. reesei* was more effective than *T. koningii* in inhibiting the development of *L. theobromae*. Based on these findings, *T. reesei* was suggested as a biocontrol strain for preventing and controlling blue stain in rubberwood.

## 1. Introduction

The rubber tree (*Hevea brasiliensis*) is an important tropical cash crop in China [1], and its primary product is natural latex. However, in recent years, due to a decline in rubber production capacity, harvested rubber trees became a valuable timber resource [2]. Unfortunately, rubberwood is highly susceptible to discoloration and decay due to its high content of sugars, proteins, and other substances. Fungal infestations in rubberwood are primarily caused by decay fungi, discoloration fungi, and wood molds, with blue-stain fungi being the most problematic among the discoloration fungi. Blue-stain fungi not only infest the wood surface but can also rapidly penetrate the wood tissue, reproducing colored mycelium and causing internal discoloration [3,4].

Although wood blue-stain fungi do not cause wood decay or affect the strength of the wood, they make the wood surface blue, bluish, or even black, affecting the appearance of the wood, reducing the value of the wood and wood products and causing great economic losses in serious cases. Wood blue-stain fungi use the cell cavity inclusions as nutrients and cause color change, which is the most typical way of color change. The blue-stain fungi’s dark mycelium and its secretion of pigment spread through the pore mouth from one cell to the neighboring cells. The sapwood wood rays and thin-walled tissue cells are rich in the nutrients needed by the mycelium and are easily discolored. Blue-stain fungi grow and reproduce at an optimum temperature of 25–35 °C; below 7 °C or above 40 °C, blue-stain fungi stop growing and reproducing. Most of the blue-stain fungi have an optimal wood moisture content of 35–60%. If the wood moisture content is lower than 20% or higher than 100%, it can inhibit the growth and reproduction of blue-stain fungi [5].

Biological control of wood discoloration in foreign countries began in the 1940s. Mae S. Chidester of the U.S. Forest Products Laboratory was the first to propose the possibility of controlling wood discoloration by biological means, but due to the lack of relevant experimental work and the absence of social demand in this area, the biological control technology did not receive further attention or research. In Canada, Forintek isolated a strain of Cartapip 97^TM^, which had been patented, and conducted field trials in Alberta and British Columbia with good results. Colleen et al. in New Zealand further pointed out that the use of soil microorganisms mixed populations with appropriate nutrients and adjuvants could also effectively prevent the spread of cyanobacteria on radiata pine logs [5]. At that time, three main approaches were employed to prevent wood discoloration: physical, chemical, and biological protective measures [6,7,8]. Regarding inhibitory strains, several colorless fungi, such as *Trichoderma*, *Penicillium*, and *Aspergillus*, were identified and shown to effectively control tree discoloration bacteria, brown rot, and white rot [5].

In the 1970s, Chinese researchers began studying wood discoloration issues, with particular focus on the problems of discoloration and decay in rubberwood during use [9]. Liu Yuan et al. [10] selected the *T. koningii* strain (6111-ET) isolated from rubberwood and obtained a crude assay of the enzyme activity with an antagonistic effect on blue-stain fungi *L. theobromae* through an induced culture. This provided scientific references for the mechanism of wood blue-stain fungi inhibition, the industrial production of chitinase biocontrol, and its application in wood blue-stain biocontrol by *Trichoderma* biocontrol fungi. It offered a scientific reference for the mechanism of inhibition of wood blue-stain fungi by wood mold biocontrol fungi, the industrial production of chitinase biopreparations, and its application in wood blue-stain biocontrol. According to Sun [11], the colorless strain of *Ophiostoma* spp. was found to be a good chemical agent in indoor environments, which not only preserved wood temporarily but also had a strong preventive effect under field conditions. Due to the serious pollution of the environment caused by traditional chemicals, domestic scholars began to study new low-toxicity agent formulations and treatment techniques, and Fang [12] and others summarized the progress of research on this type of new wood preservative. Li [13] used a new chemical anti-blue-stain agent on rubberwood to carry out a test study of mold and mildew. The results showed that treatment with the anti-discoloration agent effectively prevented blue staining and mold on rubberwood before kiln drying, and it effectively solved the problem of rubberwood’s blue stain and mildew, with a protection period of more than 4 weeks. In Jiang’s [14] field test research on rubberwood anti-mold and anti-blue stain properties, two new low-toxicity chemical fungicides were applied, with sodium pentachlorophenol being used for comparison. The results showed that F2 could keep rubberwood panels air-drying for the basic 12 days without mildew or blue staining, though the prevention and control of white mold was poorer. The F1/F2 mixture could keep rubberwood lumber air-drying for 15 days without mold or blue staining, and increasing the concentration of F1/F2 could extend the wood’s air-drying period.

Biological control is a recently developed method for preventing fungal bluing by inoculating fresh logs or wood chips with albino strains of fungi that competed for nutrients and prevented other colored strains from invading. Endophytic fungi in trees are an integral part of the natural microbial environment and have a close, mutually beneficial relationship with their host plants [15,16]. They promote the growth of the host plant and improve its resistance to diseases [17] while also preventing the growth of pathogenic fungi by competing for nutrients and space [18,19,20] and producing antibiotics and hydrolytic enzymes to inhibit the growth and ultimately kill pathogenic fungi [21]. Given the potential of plant endophytic fungi to resist diseases, pests, and tumors [22,23], it is a significant and worthwhile scientific endeavor to investigate whether the endophytic fungi of rubber trees can act as albino fungi to control the bluing condition of rubberwood.

In this study, we extracted blue-stain fungi from rubberwood and isolated for endophytic fungi with a strong inhibitory effect on blue-stain fungi to use as biocontrol strains. We then studied the antagonism of these biocontrol strains against blue-stain fungi and evaluated their inhibitory effect. This research has practical value and guiding significance for the development of biocontrol strategies for bluing in rubberwood [24].

## 2. Materials and Methods

### 2.1. Specimens

The wood used in this study originated from fresh rubber trees without discoloration in Xishuangbanna. Based on a review of the literature, it was known that rubber trees typically underwent bluish discoloration within 1 to 3 days after being cut down. Freshly felled rubberwood was transported to the laboratory, where it was allowed to naturally undergo blue discoloration for subsequent experiments.

### 2.2. Isolation of the Blue-Stain Fungus

Potato dextrose agar medium (PDA) was prepared, and plates, medium, and forceps were autoclaved. Petri dishes were sterilized by passing them over the flame of an alcohol lamp. A small opening was created, and 15 to 20 mL of culture solution was poured into each dish. The dishes were then exposed to UV light for 20 min to sterilize the culture medium. Once the medium had solidified, blue-discoloring wood chips were inoculated onto the medium, and the dishes were sealed with Parafilm and labeled. All Petri dishes were placed in a sealed bag and incubated in a constant temperature and humidity chamber (temperature: 28 °C, humidity: 68%). Upon observing the growth of mycelium, uncontaminated colonies with darker colors were selected and transferred to fresh medium. This process was repeated 2 to 3 times to obtain purer strains, which were then expanded for further use. The extracted strains were then identified.

### 2.3. Isolation of Endophytic Fungi

A round piece approximately 2 cm in diameter was cut from non-blue-stained rubberwood, and 12 additional cubes measuring 1 cm × 1 cm × 1 cm were cut from the cut surface. These samples were soaked in sterile water for 2 h. In an ultraclean bench, the samples were surface sterilized with 70% ethanol for 5 min and 3% hydrogen peroxide for 7 min, followed by rinsing with 50 mL of sterile water 3 times. The treated wood blocks were then scribed on a sterile plate using tweezers, and four blocks were placed in another sterile plate, which was labeled. The plates were observed for the presence of mycelial growth on the scribed plates and the wood blocks [25].

### 2.4. The Blue-Stain Fungus and Endophytic Fungi Identification

#### 2.4.1. Molecule Identification

The principle of identification was that the internal transcribed spacer (ITS) sequences of fungi were highly conserved among different species, and the sequences were not the same among different species. Therefore, the sequences were amplified by polymerase chain reaction (PCR), sequenced, and compared with the known sequences in GenBank, which allowed for the determination of the species of the fungi, and the fungi were classified into genera or species. Ten-day-old fresh mycelia were used for DNA extraction using the TSINGKE Plant Fungus Genomic DNA Extraction Kit. PCR processing was used to amplify the ITS gene (internal transcribed spacer 1, 5.8S ribosomal RNA gene, and internal transcribed spacer 2) using primers ITS1/ITS4 [26]. The PCR amplification followed Du et al.’s method [27], and the total volume of PCR mixtures for amplification was 25 μL, with 94 °C for 3 min, (94 °C for 30 s, 55 °C for 50 s, 72 °C for 90 s) × 35 cycles, 72 °C for 10 min, and a final step at 4 °C. Finally, the PCR products were purified and sequenced by Qinke Biotech Co., Kunming, China [16].

After obtaining the sequence data, the sequencing results were transferred to the National Center of Biotechnology Information(NCBI) nucleic acid sequence database, and BLAST, a sequence comparison tool, was used for homology comparison to select fungal strains with more than 99% homology with the tested strains and with a clear taxonomic status. A phylogenetic tree was constructed using the neighbor-joining method of MEGA11 software, and a confidence test was performed by a bootstrap analysis. The number of data sets was 1000 times.

#### 2.4.2. Morphological Identification

The isolated and purified endophytic fungi and blue-stain fungi were inoculated in PDA medium and placed in a constant temperature incubator at 28 °C for cultivation; daily observation and recording of colony morphology, color, texture, the presence or absence of pigmentation, and other characteristics of the strains were recorded, and mycelium, conidium peduncle, and conidium were observed under a light microscope; the taxonomic identification was based on the *Handbook of Fungal Identification* [28] and combined with the relevant literature for morphological identification.

### 2.5. Methods and Indicators of Antagonism Tests

*L. theobromae* and *T. koningii*, which had been previously cultured, were prepared on the workbench. Using a sterilized hole punch, a certain number of 5 mm discs were punched from the original plates. Using forceps, discs of *L. theobromae* (right) and *T. koningii* (left) were placed on the same freshly prepared medium, maintaining a distance of approximately 4 cm between the two. The plates were incubated at 28 °C in a constant temperature and humidity chamber and were observed for about one week. When the mycelia had covered the plates, the area covered by each was calculated. The resistance of the biocontrol strains to the blue-stain fungi was assessed by comparing the percentage of blue-stain fungi coverage on the plates [29]. A similar treatment was applied to *L. theobromae* and *T. reesei*. The control group was *L. theobromae* inoculated on the right side of the medium, while the left side was not inoculated with anything.

On day 7 after the endophytic fungus were inoculated, the blue-stain fungi radial growth in each plate was recorded using a caliper. The percentage inhibition of radial growth (PIRG) was calculated using Equation (1) adapted from Korsten et al. [30]:

PIRG (%) = [(r1 − r2)/r1] × 100(1)

where

r1 = the colony radius of the blue-stain fungi in control and

r2 = the colony radius of the blue-stain fungi in treatment.

The interaction assays were scored according to Bell’s scale [29], where

R1 = Blue-stain fungi grew all over the PDA;

R2 = Blue-stain fungi occupied more than two-thirds of the PDA;

R3 = Blue-stain fungi occupied one-third to two-thirds of the PDA;

R4 = Blue-stain fungi occupied less than one-third of the PDA;

R5 = Blue-stain fungi did not grow on the PDA.

### 2.6. Determination of Chemical Components and Structure

Discolored and non-blue-stained wood chips were subjected to polyethylene glycol (PEG) embedding treatment. The samples were first saturated with water and then sequentially soaked in 20%, 40%, 60%, 80%, and 100% PEG solutions. Following this, the wood was fixed in 100% PEG solution with a molecular weight of 2000. Thin sections of approximately 6–20 μm thickness were obtained using a microtome, sealed with drops of glycerol, and observed under a biomicroscope using normal light, polarized light, and fluorescence. The cell walls of the wood exhibited birefringence due to the crystalline zones formed by the ordered arrangement of cellulose molecules, which appeared brightly under polarized light. Phenolic substances in the lignin of the cell walls could produce autofluorescence, and the intensity of this autofluorescence could be observed under fluorescence microscopy. These observations allowed for a qualitative assessment of cellulose and lignin degradation [31].

Fourier-transform infrared spectroscopy (FTIR) analysis was performed on rubberwood wood chips before and after discoloration using the potassium bromide press method. The wood chips were initially crushed using a pulverizer and then ground into a fine powder with the addition of liquid nitrogen. The powder was baked until a constant weight was achieved. Both discolored and non-blue-stained wood powders (1.5 mg each) were mixed with 200 mg of potassium bromide in an agate mortar, ground, and pressed into tablets. The infrared spectral data were measured by an infrared spectrometer in the wavelength range of 500–4000 cm⁻^1^, and the results were plotted using Origin 2021 software [32].

Non-blue-stained and discolored rubberwood chips were analyzed by X-ray diffraction (XRD). The wood chips were ground into a fine powder, and an appropriate amount of the sample was flattened and compacted on a slide. The data were measured by an X-ray diffractometer and characterized in the range of 10–80°, and the results were plotted using Origin 2021 software.

Normal rubberwood without blue discoloration and rubberwood specimens inoculated with the two types of fungi for one week and three weeks, respectively, were selected and prepared into wood sections, each with a size of less than 1 cm × 1 cm × 1 cm. Scanning electron microscopy–energy dispersive spectroscopy (SEM–EDS) was used to examine the distribution of the discolored mycelium [33,34].

## 3. Results

### 3.1. Results of the Identification of Blue-Stain Fungi and Endophytic Fungi

**Figure 1:**
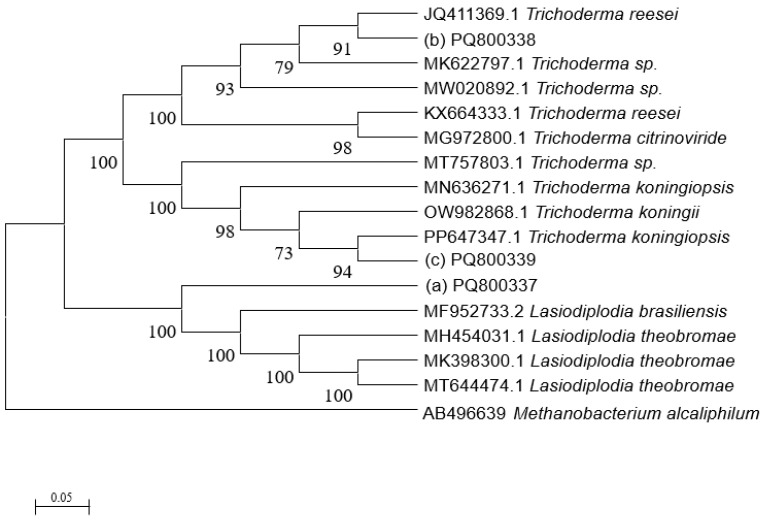
Neighbor-joining phylogenetic tree of three fungal isolates (outgroup *Methanobacterium alcaliphilum*).

According to the sequencing results, the obtained ITS gene sequencing results were inputted into the NCBI database for BLAST homology comparison, and the sequences of strains with greater homology were selected, and a sequence comparison was performed using MEGA11 software to determine the strain genus and ultimately construct the strain phylogenetic tree [35]. The observation and molecular identification (Table 1 and Figure 1) were carried out through morphological characterization of the fungi. A total of three species of fungi were identified. Table S1 shows the colony characteristics, conidial peduncles, and conidia of the three strains. The identification results are shown below:

(a) *L. theobromae*

In the PDA medium, colonies were initially white or light grey, later becoming bluish-black or all black, woolly, or cottony. Mycelium was usually colorless or light grey, elongated and non-septate, and formed tighter clusters. Conidiophores were usually erect or slightly curved, elongated, and colorless or yellowish. Conidia were spherical or ellipsoidal, about 10–20 µm long and 4–6 µm wide, and usually colorless to pale yellow or light brown.

(b) *T. reesei*

In the PDA plate media, colonies were usually white or creamy white, with the surface color changing to yellow over time and a musty or characteristic fermentation odor being present when cultured. Mycelium was white, grew rapidly and densely, and branched frequently. The mycelium was non-septate, hollow, tougher, and irregularly branched. Conidiophores were short, hyaline, and erect. Conidia were colorless, about 3–5 μm in diameter, and oval or ovoid in shape.

(c) *T. koningii*

In the PDA plate medium, the colonies were white and gradually changed to green or yellow. Mycelium was white to light green; grew rapidly; branched frequently; was non-septate, hollow, in slender chains or branches; and formed a dense mycelial network on the medium. Conidiophores were short, erect, and hyaline or slightly yellowish. Conidia were green or yellowish-green in color, with a diameter of 3–5 μm and a round or oval shape.

### 3.2. Analysis of the Antagonistic Effect

In this experiment, the strains of *T. reesei* and *T. koningii* were selected to carry out antagonistic experiments with the blue-stain fungus *L. theobromae*. Cumulative observations were performed for about 7 days, and when the blue-stain fungus *L. theobromae* and the biocontrol strains had grown to fill the entire Petri dish (Table 2), the area occupied by each of them, as well as the inhibition rate, were calculated, and the inhibition grade was classified (Table 3). The radius of a Petri dish was 4.5 cm, and the total area was 63.585 cm^2^. Through observation and calculation, it was observed that the inhibition effect of *T. reesei* on *L. theobromae* was better, while the inhibition effect of *T. koningii* was weaker.

### 3.3. Polarized Light and Fluorescence Microscopy Analysis

**Figure 2:**
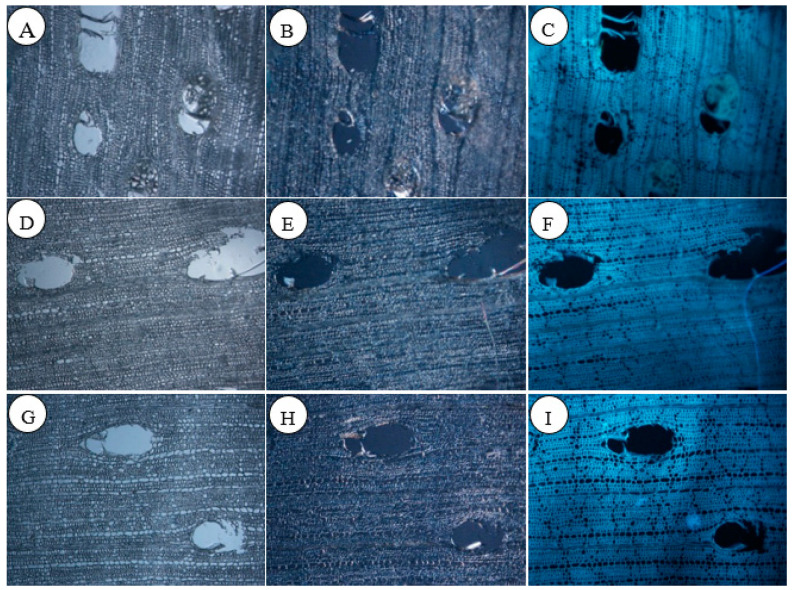
(**A**–**C**) are normal light, polarized, and fluorescent pictures of cross-sections of non-blue-stained rubberwood; (**D**–**F**) are normal light, polarized light, and fluorescence pictures of the cross-section of rubberwood of *T. koningii* at three weeks of inoculation; (**G**–**I**) are normal light, polarized light, and fluorescence pictures of the cross-section of rubberwood of *T. reesei* at three weeks of inoculation (10×).

A comparison was performed between ordinary light, polarized light, and fluorescence imaging of the cross-sections of non-blue-stained rubberwood and rubberwood inoculated with fungi. Cellulose, the structural material constituting the cell wall, appeared brightly and distinctly under the polarized light microscope. From the comparison of the polarized images of rubberwood thin sections inoculated with fungi versus non-blue-stained sections, it could be observed that cellulose degradation was minimal in the inoculated rubberwood thin sections. Lignin, a major component of the cell wall matrix, was not uniformly distributed in the wood but fluoresced distinctly under the fluorescence microscope. Similar to cellulose degradation, the comparison of the polarized images of the inoculated rubberwood thin sections and the non-blue-stained images revealed minimal lignin degradation. The comparison of the polarized and fluorescent images of the rubberwood thin sections inoculated with fungi and the non-blue-stained thin sections showed no significant differences, indicating that the degradation of cellulose and lignin by these two fungi was not substantial (Figure 2).

### 3.4. FTIR Analysis

**Figure 3:**
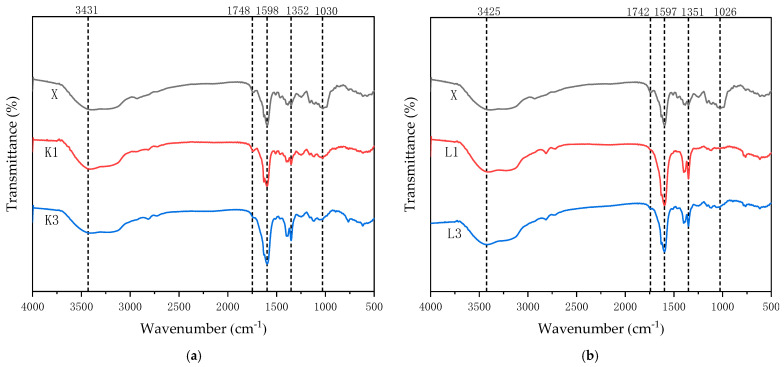
(**a**) shows the infrared spectra of non-blue-stained rubberwood flakes and rubberwood flakes inoculated with *T. koningii*; (**b**) shows the infrared spectra of non-blue-stained rubberwood flakes and rubberwood flakes inoculated with *T. reesei*. X is non-blue-stained rubberwood; K1 is one week of rubberwood inoculated with *T. koningii*; K3 is three weeks of rubberwood inoculated with *T. koningii*; L1 is one week of rubberwood inoculated with *T. reesei*; L3 is three weeks of rubberwood inoculated with *T. reesei*.

Fourier-transform infrared spectroscopy (FTIR) was mainly used to analyze the structure of wood composition and changes in the chemical composition of the wood [36]. The trends of infrared spectral spectra of rubberwood lamellae versus non-blue-stained rubberwood lamellae at one week and three weeks after inoculation with *T. koningii* are shown in Figure 3a. The trends of infrared spectral spectra of rubberwood lamellae and non-blue-stained rubberwood lamellae in the first and third weeks after inoculation with *T. reesei* are shown in Figure 3b. Changes in cellulose, hemicellulose, and lignin were assessed on the basis of changes in chemical bonding energy at the absorption peak.

As could be seen from Figure 3a, compared with the original rubberwood slices, the infrared spectral spectra of rubberwood slices infested with *T. koningii* for one week and three weeks had similar trends of changes, with a larger value of the intensity of the absorption peak at 3431 cm⁻^1^ and a broad peak, which was mainly caused by the hydroxyl O-H telescopic vibration of cellulose and which originated from the contribution of the main components of the wood. The cause of the peak at 1748 cm⁻^1^ was through the carbonyl C=O stretching vibration, which was closely related to the content of hemicellulose [37]. For the absorption peak at 1598 cm⁻^1^, it was attributed to the lignin benzene ring backbone stretching vibration. The absorption peak at 1352 cm⁻^1^ was mostly due to the C-H stretching vibration on hemicellulose. The absorption peak near 1030 cm⁻^1^ was attributed to the stretching vibration of the ether bond in cellulose. This indicated that hemicellulose and lignin did not change significantly, while cellulose underwent weak degradation [38].

Figure 3b showed the infrared spectral spectra of rubberwood slices after one week and three weeks of infestation by *T. reesei*. The trend of change was also similar compared with that of non-blue-stained rubberwood slices. The changes of its individual absorption peaks were basically the same as those in Figure 2A, indicating that hemicellulose and lignin did not undergo obvious changes, while cellulose underwent weak degradation.

### 3.5. XRD Analysis

**Figure 4:**
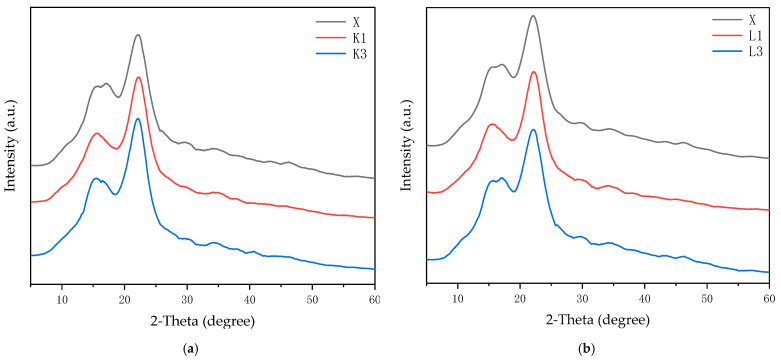
(**a**) XRD plot of non-blue-stained rubberwood and rubberwood inoculated with *T. koningii*; (**b**) XRD plot of non-blue-stained rubberwood and rubberwood inoculated with *T. reesei*. X is non-blue-stained rubberwood; K1 is one week of rubberwood inoculated with *T. koningii*; K3 is three weeks of rubberwood inoculated with *T. koningii*; L1 is one week of rubberwood inoculated with *T. reesei*; L3 is three weeks of rubberwood inoculated with *T. reesei*.

XRD was mainly used to analyze the magnitude of relative crystallinity of non-blue-stained rubberwood and rubberwood inoculated with the fungi. The X-ray diffraction patterns of rubberwood flakes infested with *T. koningii* and *T. reesei* were very similar to those of non-blue-stained rubberwood flakes, which indicated that the infestation of the two fungi had no significant effect on the regular crystalline zone of rubberwood [39], as shown in Figure 4a,b.

The relative crystallinity was calculated using the Segal method, and it was found that the crystallinity of the thin slices inoculated with both fungi had increased, which might have been attributed to the transformation of some of the amorphous and indeterminate zones in the rubberwood into crystalline zones, resulting in greater crystallinity [40]. The two diffraction peaks appearing at about 16° and 22° of the specimen were typical reflective surfaces of wood cellulose, and there were no other obvious characteristic diffraction peaks [41]. A comparison showed that after infestation by *T. reesei* and *T. koningii*, the two diffraction peaks at 16° and 22° of the rubberwood flakes had a slight decrease in the peak value, indicating that the cellulose in the rubberwood flakes infested by *T. reesei* and *T. koningii* had undergone slight degradation, which was consistent with the results of the infrared spectroscopic analysis.

### 3.6. SEM–EDS Analysis

**Figure 5:**
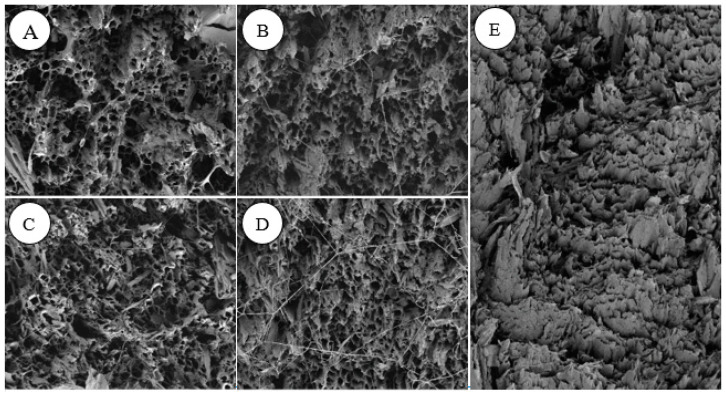
(**A**) Scanning electron micrograph of rubberwood inoculated with *T. koningii* for one week; (**B**) scanning electron micrograph of rubberwood inoculated with *T. koningii* for three weeks; (**C**) scanning electron micrograph of rubberwood inoculated with *T. reesei* for one week; (**D**) scanning electron micrograph of rubberwood inoculated with *T. reesei* for three weeks; (**E**) scanning electron micrograph of rubberwood with no discoloration.

Figure 5A,B show the scanning electron microscope picture of rubberwood inoculated with *T. koningii*. Figure 5C,D show the scanning electron microscope picture of rubberwood inoculated with *T. reesei*, and Figure 5E shows the scanning electron microscope picture of rubberwood that had not been discolored. By observing the pictures in Figure 5A–D and comparing them with those in Figure 5E, it was found that mycelium had formed in all of the rubberwoods inoculated with the fungus, while no mycelium was found in the non-blue-stained rubberwoods. It was observed that there were more mycelia in the third week than in the first week in the rubberwood inoculated with the fungus and that more mycelia had grown in the rubberwood inoculated with *T. reesei* than in the rubberwood inoculated with *T. koningii*.

**Figure 6:**
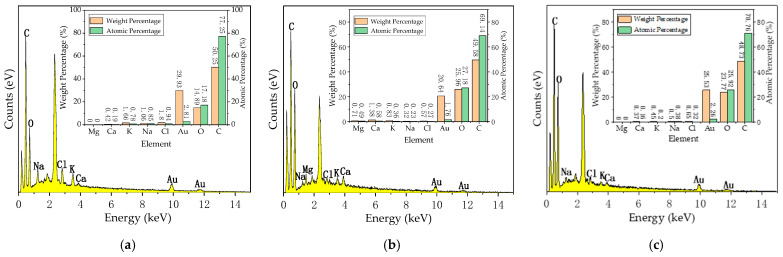
(**a**) Total spectrum of elements of rubberwood inoculated with *T. koningii*; (**b**) total spectrum of elements of rubberwood inoculated with *T. reesei*; (**c**) total spectrum of elements of non-blue-stained rubberwood.

Elemental line total spectra analysis: Figure 6a shows the elemental line total spectra of rubberwood inoculated with *T. koningii*; Figure 6b shows the elemental line total spectra of rubberwood inoculated with *T. reesei*; and Figure 6c shows the elemental line total spectra of non-blue-stained rubberwood, all of which were inoculated with the fungus in the third week. The weight percentage and atomic percentage of each element in the three samples are also shown. It could be observed that the elements C, O, Au, Cl, Na, K, and Ca are labeled in all three graphs, where the element C is more abundant. The atomic percentages of the element C for X, K3, and L3 were 77.25%, 69.14%, and 70.76%, respectively, followed by the elements O and Au, while the other elements were less abundant. The presence of the element Mg is found in Figure 6b. From the table, it can be seen that K3 and L3 contained more of the element O than X, while C, Au, Cl, Na, and K were all less abundant than in X. For the element Ca, the content in K3 was more abundant than in X and L3.

## 4. Discussion

In this study, the fungus *L. theobromae* was isolated from rubberwood in Xishuangbanna, which had been discovered by Cooke as early as 1879 and was found to be a widely distributed fungus in the tropical, subtropical, and temperate zones, with about 500 species of host plants. This group of fungi not only causes plant diseases but is also one of the key fungi responsible for the blue stain of tropical broadleaf trees [42]. The optimal temperature range for its survival is 25 to 35 °C, and the ideal temperature is 30 °C. Its mycelial growth slows down in very-high- or low-temperature environments. Its satisfactory pH interval is 5.0 to 9.0, and it is a phototrophic fungus, where light stimulates the growth of its mycelium [43]. *Trichoderma* and *Aspergillus* spp. are frequently reported as beneficial fungi in recent studies. Endophytic fungi can effectively promote plant growth and improve plant disease resistance [44]. Two endophytic fungi, *T. koningii* and *T. reesei*, were extracted from non-blue-stained rubberwood, both belonging to the *Trichoderma*. Antagonistic behavior against a wide range of phytopathogenic fungi has been observed in several species such as *Trichoderma harzianum* [45], *Trichoderma viride*, *T. koningii*, and *Trichoderma hamatum* [46].

In the field of antagonism research, Li [47] successfully isolated and characterized the strains responsible for the wood discoloration of *Betula alba* and identified a high-quality antagonistic ability of *Bacillus subtilis* B26. *B. subtilis* played an extremely important role in the biological control of plant diseases due to its excellent colonization efficacy and easy screening qualities. Zhang [29] similarly isolated and characterized the controlling strains, i.e., *Trichoderma citrinoviride* and *T. koningii*, which caused the blue stain of rubberwood and horsetail pine, and conducted an in-depth study on their biological characteristics. According to previous research and combined with their own tests, it was found that there is indeed an inhibitory effect of *T. koningii* on *L. theobromae*. As for the inhibitory effect of *T. reesei* on *L. theobromae*, no relevant articles have been published. Although many experts have conducted extensive research on the biological control of rubberwood discoloration, the effect of applying biological control methods to control rubberwood discoloration commercially have not been satisfactory. The biological control of wood discoloration in this experiment was carried out in the laboratory (PDA), and no biological control tests have been carried out in rubberwood in vivo. It is well known that the environment under natural conditions is much harsher than in the laboratory, indicating that environmental conditions should be considered in the evaluation of the efficacy of biological control.

Polarized light and fluorescence microscopy failed to show a significant effect of the inoculated fungi on the cellulose and lignin degradation of rubberwood, suggesting that these two endophytic fungi caused less structural damage to the wood. The results of infrared spectroscopy further confirmed that *T. koningii* and *T. reesei* had a weak effect on the degradation of the hemicellulose and lignin of rubberwood, showing only weak degradation of cellulose. This indicated that these two fungi could affect the blue stain phenomenon mainly through antagonistic inhibition of the growth of blue-stain fungi rather than through direct degradation of wood components [48]. The XRD analysis further supported the results of the IR spectra, indicating that cellulose in rubberwood did undergo slight degradation in the presence of *T. koningii* and *T. reesei*. This slight degradation may have been caused by the growth activity of the fungi, especially in the case of mycelial infestation of the fungi, where the structure of the rubberwood was altered to some extent. However, the degree of degradation was much lower than in the case of direct decay or pathogenic fungal infestation [49]. The presence of mycelium formation within the rubberwood inoculated with the fungus was observed by scanning electron microscopy, and more mycelium was observed in the third week than in the first week. This suggested that the growth of the fungus on the surface and inside the rubberwood gradually increased over time. In addition, rubberwood inoculated with *T. reesei* exhibited more mycelial growth compared with *T. koningii*, which may have indicated that *T. reesei* was more capable of colonizing and expanding on the surface of rubberwood [50].

In conclusion, further studies on the growth conditions and biological properties of the fungus *L. theobromae* and the extracted endophytic fungi of rubberwood blue stain are needed. In addition, in-depth studies on endophytic fungi associated with rubberwood are needed for the application and effect analysis of the extracted endophytic fungi on living standing trees in order to develop effective biocontrol agents [51].

## 5. Conclusions

In this paper, the blue-stain fungi isolated from blue-stained rubberwood was identified as *L. theobromae*. Endophytic fungi isolated from non-blue-stained rubberwood were identified as *T. koningii* and *T. reesei*. The two fungi were tested for antagonism against *L. theobromae*, and *T. reesei* was found to be more effective at inhibiting the fungus. Characterization of the chemical components suggests that the two Trichoderma isolates could inhibit the growth of the blue-stain fungus through antagonism rather than affecting the blue stain phenomenon through direct degradation of wood components.

## Figures and Tables

**Table 1:** Number, form, ITS GenBank accession number, strain name, genus, family, order, and class of strain.

Number	Form	ITS GenBank Accession Number	Strain Name	Genus	Family	Order	Class
(a)	Blue-stain fungi	PQ800337	*L. theobromae*	*Lasiodiplodia*	Botryosphaeriaceae	Botryosphaeriales	Dothideomycetes
(b)	Endophytic fungi	PQ800338	*T. reesei*	*Trichoderma*	Hypocreaceae	Hypocreales	Sordariomycetes; Hypocreomycetidae
(c)	Endophytic fungi	PQ800339	*T. koningii*	*Trichoderma*	Hypocreaceae	Hypocreales	Ascomycetes

**Table 2:** Plot of 1–7 days of inhibition of the blue-stain fungi by the biocontrol strains.

Biocontrol Strain (Left)–Blue-Stain Fungi (Right)	Biocontrol Strains and Blue-Stain Fungi Days of Inoculation			
	At One Day of Inoculation	At Three Days of Inoculation	At Five Days of Inoculation	At Seven Days of Inoculation
*T. koningii–L. theobromae*	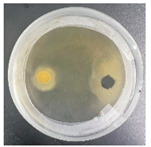	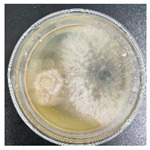	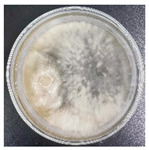	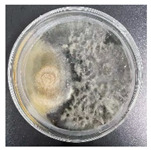
*T. reesei–L. theobromae*	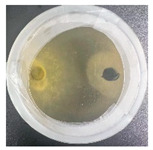	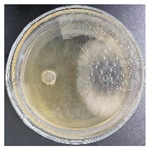	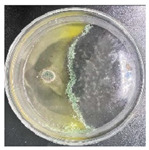	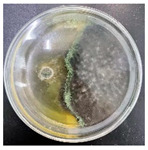

**Table 3:** Effect of biocontrol strains on percentage inhibition of radial growth (PIRG) of blue-stain fungi. Area occupied by biocontrol strain and Bell’s ranking scale [29] categories at 7 days of incubation on the PDA.

Biocontrol Strain–Blue-Stain Fungi	PIRG (%)	Area Occupied by Biocontrol Strain (cm^2^)	Bell’s Ranking Scale Categories
*T. koningii*–*L. theobromae*	23.3~24.9	15.30 ± 0.5	R2
*T. reesei*–*L. theobromae*	44.9~46.4	29.02 ± 0.5	R3

## Data Availability

Data are contained within the article and supplementary materials.

## Supplementary Materials

The following supporting information can be downloaded at: https://www.mdpi.com/article/10.3390/jof11010055/s1, Table S1 shows the colony characteristics, conidial peduncles, and conidia of the three strains.
