# Supplementary material for: Study of the Antagonism of Biocontrol Strains Against the Blue-Stain Fungus of Rubberwood
Source: J Fungi (Basel). 2025 Jan 12;11(1):55. doi: 10.3390/jof11010055 (PMC11766317; doi:10.3390/jof11010055)
Supplement: Supplementary file 1 [file jof-11-00055-s001.zip › jof-3367659-supplementary.pdf]

Table S1. Colony characteristics, Conidium peduncle and Conidium of three strains. (Under microscope Leica: 20×)

	**(a)** *Lasiodiplodia theobromae*	**(b)** *Trichoderma reesei*	**(c)** *Trichoderma koningii*
Colony characteristics	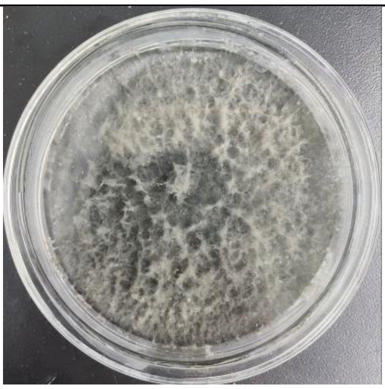	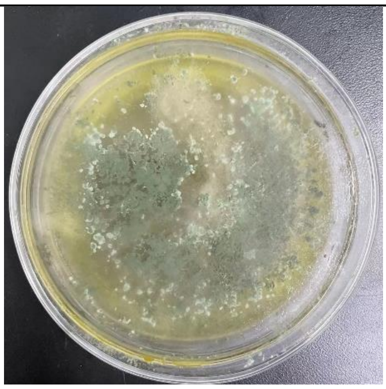	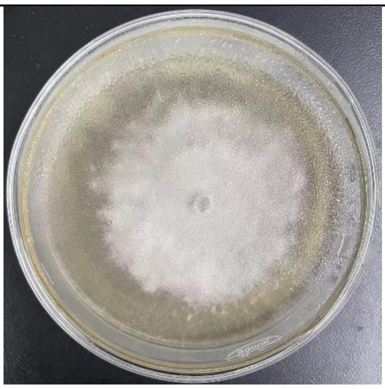
Conidium peduncle	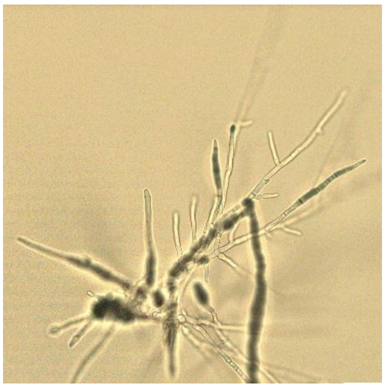	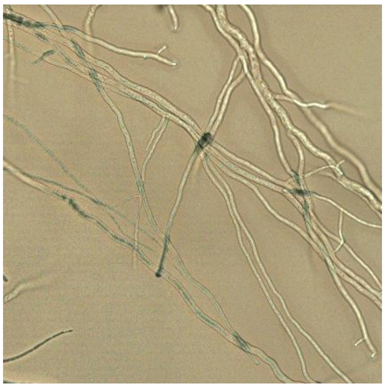	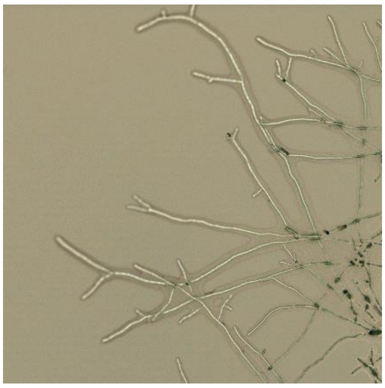
Conidium	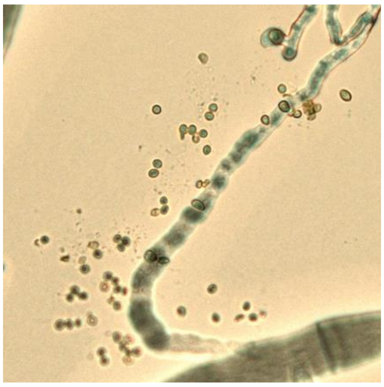	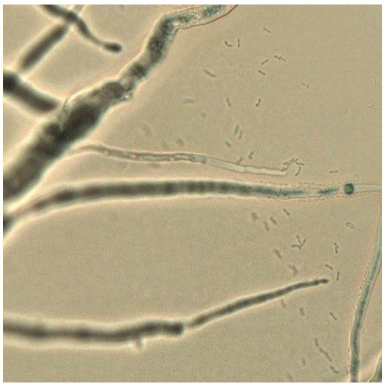	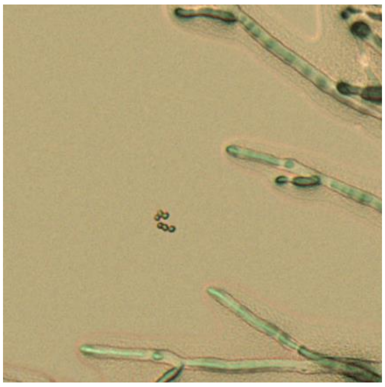
